# Association of smoking with amyotrophic lateral sclerosis: A systematic review, meta-analysis, and dose-response analysis

**DOI:** 10.18332/tid/175731

**Published:** 2024-01-18

**Authors:** Kihun Kim, Dai Sik Ko, Jin-Woo Kim, Dongjun Lee, Eunjeong Son, Hyun-Woo Kim, Tae-Jin Song, Yun Hak Kim

**Affiliations:** 1 Department of Biomedical Informatics, School of Medicine, Pusan National University, Yangsan, Republic of Korea; 2 Department of Anatomy, School of Medicine, Pusan National University, Yangsan, Republic of Korea; 3 Division of Vascular Surgery, Department of General Surgery, Gachon University Gil Medical Center, Incheon, Republic of Korea; 4 Department of Oral and Maxillofacial Surgery, School of Medicine, Ewha Womans University Medical Center, Republic of Korea; 5 Division of Respiratory and Allergy, Department of Internal Medicine, Pusan National University Yangsan Hospital, Yangsan, Republic of Korea; 6 Department of Neurology, Pusan National University Yangsan Hospital, Yangsan, Republic of Korea; 7 Department of Neurology, Seoul Hospital, Ewha Womans University College of Medicine, Gangseo-gu, Republic of Korea

**Keywords:** smoking, amyotrophic lateral sclerosis, systematic review, meta-analysis, dose-response analysis

## Abstract

**INTRODUCTION:**

Amyotrophic lateral sclerosis (ALS) is a progressive neurodegenerative disorder primarily affecting the voluntary motor nervous system. Several observational studies have provided conflicting results regarding the association between smoking and ALS. Therefore, our objective was to investigate this association through a systematic review, meta-analysis, and dose-response analysis.

**METHODS:**

On 16 January 2023, we initially extracted records from medical databases, which included Medline, Embase, Web of Science, Scopus, and ScienceDirect. We included case-control and cohort studies as eligible studies. Subgroup analyses were performed based on sex, study design, and current smoking. Restricted cubic-spline analysis was utilized to assess the dose-response relationship between smoking (pack-years) and ALS.

**RESULTS:**

Twenty-eight case-control and four cohort studies met the inclusion criteria. The unadjusted OR for the overall association between smoking and ALS was 1.14 (95% CI: 1.06–1.22, I^2^=44%, p<0.001), and the adjusted OR (AOR) was 1.12 (95% CI: 1.03–1.21, I^2^=49%, p=0.009). Subgroup analysis revealed a more pronounced association among current smokers, with an AOR of 1.28 (95% CI: 1.10–1.49, I^2^=66%, p<0.001) and AOR of 1.28 (95% CI: 1.10–1.48, I^2^=58%, p=0.001). In the dose-response analysis, the non-linear model revealed an inverted U-shaped curve.

**CONCLUSIONS:**

Our study provides evidence of a positive relationship between smoking and the risk of ALS. To mitigate the risk of developing ALS, discontinuing smoking, which is a modifiable risk factor, may be crucial.

**TRIAL REGISTRATION:** The study was registered in PROSPERO.

**IDENTIFIER:** CRD42023388822

## INTRODUCTION

Amyotrophic lateral sclerosis (ALS) is a debilitating neurodegenerative disorder that primarily affects the voluntary motor nervous system. It is characterized by a progressive weakening and spasticity of the affected regions, with symptoms gradually spreading from the initial site(s) of onset^1^. Given the absence of effective therapeutic interventions for ALS and its substantial impact on individuals and society, addressing this condition is an urgent global concern^2,3^.

Both genetic and environmental factors have been identified as contributors to the risk of developing ALS. In terms of genetic risk factors, several genes, namely SOD1, FUS, TDP43, and C9orf72, have been associated with the occurrence of ALS^4-6^. As for environmental factors, various factors such as mercury, lead, pesticides, solvents, head trauma, electric shock, and lower body mass index have been suggested as potential risk factors for ALS^7^.

While smoking is a significant risk factor for various diseases and is well-established as the primary preventable cause of death, the relationship between smoking and ALS has been studied extensively, with varying and inconclusive findings in the existing literature. While some previous studies have indicated a weak positive relationship between smoking and ALS, others have found no significant association^7,8,9^.

As such, there is a clear demand for comprehensive investigations, including meta-analyses, to establish a dose-response relationship and gain a more thorough comprehension of the potential causal link between smoking and ALS. Therefore, our objective was to examine the association between smoking and ALS by conducting systematic reviews and dose-response meta-analyses of relevant observational studies.

## METHODS

### Protocol and registration

The protocol of this study was registered in PROSPERO and conducted in accordance with the methods described in the preferred reporting items for systematic reviews and meta-analyses (PRISMA) guidelines^10,11^.

### Information source and search strategy

On 16 January 2023, a systematic literature search was performed using various medical databases such as Medline, Embase, Web of Science, Scopus, and ScienceDirect to identify relevant published articles. In order to establish search strategies for each database, the primary focus was placed on utilizing the MeSH term and entry terms for: ‘smoking’, ‘amyotrophic lateral sclerosis’, ‘case-control study’, and ‘cohort study’. The final search strategies, which are detailed in Supplementary file Table 1 with a comprehensive description of the search techniques used for each database, were determined by consensus among all the authors. The articles that were incorporated in the previous meta-analyses were also obtained by conducting a bibliographic search of the references cited within the articles^7,12^. Google and Google Scholar were utilized for a search of grey literature, and the reference lists of pertinent publications were scrutinized to ascertain the inclusion of any missing records.

**Table 1:** Characteristics of included studies*

	*Case-control studies*										
*No.*	*First Author Year*	*Location*	*Participants*	*Period of recruitment*	*Controls*	*Case ascertainment*	*DC*	*Smoking status*	*Cases Mean age (SD)*	*Controls Mean age (SD)*	*Matching*
1	Kondo 1981	Japan	158/158	1973	Community/ hospital	Neurology clinic	NS	Yes/No	NS	NS	Age, sex, residence
2	Provinciali 1990	Ancona Italy	77/80	1979–1987	Other neurological diseases	Neurology clinic	NS	10–30 cigarettes/day	59 (8)	57 (9)	Age, sex, regional origin, life-style, cultural background
3	Savettieri 1991	Palermo Italy	46/92	NS	Friends/neighbors	Neurology clinic	NS	Yes / No	NS	NS	Age, sex, residence, socioeconomic status
4	Vinceti 1997	Reggio Emilia Italy	16/39	NS	Community	ALS clinic	EEC	Yes/No	65.9 (14.0)	64.4 (12.9)	Age and sex
5	Nelson 2000	Washington State USA	161/321	1990–1994	Community	Multiple sources	NS	Never/ever/ former/ current	61.4 (1.0)	61.7 (0.7)	Age and sex
6	Qureshi 2006	Boston USA	95/106	1998–2002	Friends/relatives	ALS clinic	EEC	Yes/No	54.4 (13.1)	52.5 (14.9)	Age and sex
7	Sutedja 2007	Utrecht Netherlands	364/392	2001–2005	Friends	ALS clinic	EEC	Never/former/ current/	60.2 (11.7)	60.0 (10.9)	Age and sex
8	Fang 2009	New England USA	109/253	1993–1996	Community	Neurology clinic	EEC	0/1–10/11–30/31+(pack-years)	NS	NS	Age, sex, residence
9	Okamot 2009	Tokai Japan	153/306	2000–2005	Community	Neurology clinic	EEC	Non-smoker/ current	63.7 (9.2)	63.4 (10.6)	Age, sex
10	Alonso 2010	UK	1143/11371	1990–2008	GPRD database	GPRD database	NA	Never/former/ current/non-heavy/ heavy	67.4 (12.5)	67.1 (12.5)	Age, sex, practice, year of enrolment
11	Beghi 2010	EURALS Consortium (Italy, UK, Ireland)	61/112	NS	Community	ALS registries	EEC	Yes/No	63.7 (NS)	62.3 (NS)	Age and sex
12	Furby 2010	Brittany France	108/112	2006–2008	Hospital (orthopedic service for minor trauma)	Neurology clinic	EEC	Non-smoker/ former/current/ pack-years	68 (18.0)	65 (18.0)	Age and sex
13	Schmidt 2010	USA	241/597	2003–2007	US army veterans	US army veterans ALS registry	EEC	Never/former/ current	62.4 (10.3)	61.7 (10.6)	Age, sex, use of veteran affairs health care
14	Das 2012	India	110/240	2008–2011	Community	Neurology clinic	rEEC	Non-present/ present	NS	NS	Age and sex
15	Moreau 2012	Nord Pas de Calais County France	102/408	2003–2009	Community	ALS clinic	EEC	Never/former/ current	NS	NS	Age and sex
16	Yu 2014	Michigan USA	66/66	NS	Community	ALS clinic	rEEC	Never/former/ current	NS	NS	Age and sex
17	Malek 2015	Pittsburgh and Philadelphia USA	66/66	2008–2010	Outpatient hospital controls	ALS clinic	EEC	Never/ever	57.1 (13.2)	56.4 (13.5)	Age, sex, race
18	Harwood 2016	Northern England UK	175/317	2009–2013	Community	Hospital/community	rEEC	Non-smoker/ex-smoker/ current	64 (NS)	65 (NS)	Age and sex
19	Nagel 2017	South-West Germany	289/506	2010–2014	Community	ALS registry Swabia	rEEC	Never/ever	65.7 (10.5)	66.3 (9.8)	Age and sex
20	Seelen 2017	Netherlands	917/2662	2006–2013	Community	Multiple sources	rEEC	Non-current/ current	63.5	63.5	Age and sex
21	Bjornevik 2019	USA	275/549	1976–2012	Cohort	5 Cohort	rEEC	Never/past/ current	64.6 (7.2)	64.6 (7.2)	Age, sex, cohort, fasting status, time of blood draw
22	Chen 2019	New Zealand	321/605	2013–2016	Community	ALS registry and hospital discharge records	NS	Never/ ex-smoker/ current	NS	NS	Age and sex
23	Lian 2019	China	123/239	2013–2016	Community	Hospital	EEC	Never/former/ current	53.2 (9.6)	53.0 (11.1)	Age and sex
24	Visser 2019	Euro-MOTOR consortium (Netherlands, Ireland, Italy)	1577/2922	2011–2014	Community	Multiple sources	rEEC	Never/former/ current	NS	NS	Age, sex, residence
25	Opie-Martin 2020	UK	202/200	2008–2013	Community	MNDA Epidemiology study	EEC	Never/former/ current	63.1 (10.53)	64.5 (10.52)	Age and sex
26	Bear 2021	USA	127/127	2018–2020	Community	National ALS Registry	NS	Never/ever/ current	NS	NS	Age, sex, residence
27	Peters 2021	Europe	107/319	1993–1999	Cohort	EPIC cohort	NS	Never/former/ current	60.5 (NS)	60.4 (NS)	Age, sex, study center
28	Magid 2022	USA	3714/18570	2006–2013	Cohort	Centers for Medicare and Medicaid Services (CMS)	EEC	Yes/No	75.7 (5.7)	75.7 (5.8)	Age, sex, enrollment length, residence
	***Cohort studies***										
	***First Author Year***	***Location***	***Participants***	***Period of recruitment***	***Average follow-up***	***Case ascertainment***	***DC***	***Smoking status***		***Age (range)***	***Adjustment***
29	Fang 2006	Sweden	160/280558	1978–1983	19.6 years	Inpatient register	NS	Non-tobacco use/former/ current		41 (NS)	Age, residence
30	Gallo 2009	Europe	116/505355	1991–2001	8.9 years	Death certificates	NA	Never/former/ current		51 (NS)	Age, sex, education level, study center
31	Wang 2011	USA	816/1119080	1986–2005	7–28 years	US NDI/selfreport	NS	Never/ever/ former/ current		NS	Age, sex, body mass index, physical activity, education level
32	Doyle 2012	UK	752/1319360	1981–2008	9.2 years	ICD-10	NS	Never/past/ current		56	Region, socioeconomic status, year of birth, body mass index, use of hormone replacement therapy, smoking, alcohol use, as appropriate

* Full references are given in Supplementary file Table 4. DC: Diagnostic criteria. ALS: amyotrophic lateral sclerosis. EEC: El Escorial Criteria. NA: not applicable. NS: not specified. NDI: National Death Index. rEEC: revised El Escorial Criteria (Airlie House Criteria). SES: socioeconomic status.

### Eligibility criteria

The PICO framework utilized in this study for the precise collection of relevant evidence is as follows:

- P (Population): People of any gender, age, or ethnicity, with available information regarding their smoking status and diagnosis of ALS, were included without any restrictions.
- I (Intervention): Smokers (current smokers and former smokers).
- C (Comparison): Non-smokers.
- O (Outcome): Odds ratio of ALS in smokers compared to non-smokers.

We selected case-control and cohort studies that included information for smoking and onset of ALS. In case of data source duplication, only articles with the largest sample size were selected. Studies that did not clearly define the control group were excluded. For case-control study selection, only those studies were included where potential confounding factors, such as sex and age, were matched. Motor neuron diseases other than ALS, such as primary lateral sclerosis, progressive bulbar palsy, and spinal muscular atrophy, were excluded from the analysis. Animal and in vitro studies, as well as review articles, cross-sectional studies, case series, abstracts, and case reports, were excluded from the analysis. There were no restrictions imposed by the study on the age of the patients, the language used, or the year of publication.

### Study selection

The corresponding author conducted a review of the initial records extracted from the search database to assess their relevance and validity. The first authors independently performed both initial screening and full-text review, and also conducted a bibliographic review of the included studies. The eligibility of the case-control and cohort studies included in previous meta-analyses was also reassessed for inclusion^12,13^. The grey literature search was also conducted by the first authors. Any discrepancies with regard to the inclusion of articles were resolved through discussion among all authors.

### Data extraction

The information from the included studies was extracted independently by the first authors through a full-text review. The types of information included the first author’s name, publication year, location, age of participants, number of participants, period of recruitment, diagnostic criteria, smoking status, and matched variables.

### Assessment of risk of bias

The potential risk of bias in the cohort and case-control studies that were included was evaluated by utilizing the Newcastle-Ottawa Scale, which is among the most commonly utilized instruments for determining risk of bias in observational research^13^. The comprehensive assessment was conducted using three domains (selection, comparability, and outcome) consisting of eight items and rated as ‘good’, ‘fair’, or ‘poor’ quality. The first authors carried out the assessments independently, and then the corresponding author reviewed them. In case of any disagreements in the evaluations, the authors resolved them through discussions.

### Effect measures

Unadjusted (ORs) and adjusted odds ratios (AORs) with 95% confidence intervals (CIs) were extracted in the included studies. If the odds ratio was not reported, it was calculated using the 2×2 contingency table. For cohort studies reporting relative risk, the Zhang and Yu^17^ method was used to convert it to an OR.

### Data synthesis and subgroup analyses

We assessed the heterogeneity of pooled effect measures using the I^2^ statistic classification proposed by Higgins et al.^15^. If the heterogeneity of the integrated results was <50%, it was considered low, and if ≥50% it was considered considerable heterogeneity. To obtain a pooled odds ratio for categorical data, we utilized the inverse variance method. We employed a random-effects model regardless of heterogeneity to accommodate the varying study designs included in the analysis. The meta-analysis was performed using Review Manager 5.4, a software program developed by Cochrane, and visualized the pooled odds ratios using forest plots. Subgroup analyses were conducted for several factors, including sex, study design (case-control, and cohort), and current smokers.

### Dose-response analysis

To perform a dose-response analysis between smoking and ALS, we used restricted cubic-spline analysis for studies containing available pack-years information on smoking^16^. To evaluate linearity in the dose-response relationship, a Wald test was conducted on three dose categories across four notes (5, 35, 65, 95 percentile) by segmenting smoking (pack-years). A dose-response graph was generated using the STATA 13 software to visually represent the association

### Publication bias

Funnel plots were utilized to quantitatively evaluate the possibility of publication bias, generated through the STATA 13 program. Egger’s regression test, executed with the STATA 13 software, was employed to determine the statistical significance of any detected publication bias.

### Certainty assessment

Grading of recommendations, assessment, development, and evaluations (GRADE) methodology was employed to evaluate the certainty of evidence for the primary outcome, which categorizes the quality of evidence as high, moderate, low, or very low, based on five essential domains (study limitations, directness, consistency, precision, and reporting bias) as well as three supplementary domains (dose-response relationship, plausible confounding factors that could decrease the observed effect, and strength of association)^17,18^.

## RESULTS

### Study selection process

**Figure 1:**
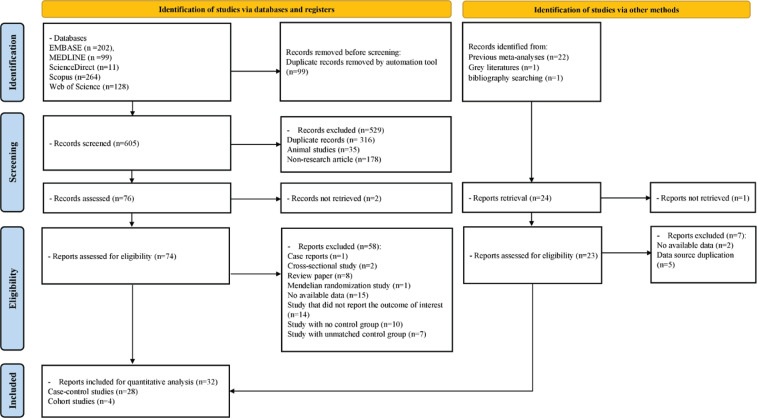
PRISMA flow diagram for systematic reviews which included searches of databases, registers, and other sources

A search was conducted in five databases using a predefined search strategy. A total of 605 records were screened after removing 99 duplicate records using deduplication tools within the databases. Of these, 314 duplicate records, 35 animal studies, and 178 non-research articles were excluded, leaving 78 records for initial screening. Two records could not be retrieved, and 76 articles underwent full-text review. In addition, 23 articles were identified through grey literature searching, bibliography reviews, and review of studies included in previous meta-analyses. Finally, 32 studies, including 28 case-control studies and four cohort studies, were selected for systematic review and meta-analysis. Other articles were excluded if they were review articles, Mendelian randomization studies, case reports, cross-sectional studies, or they lacked available data, failure to report the outcome of interest, and lack of a control group or unmatched control group. The excluded studies are presented in Supplementary file Table 5, along with their respective reasons. A PRISMA diagram that outlines the process of selecting studies for inclusion is presented in Figure 1.

### Characteristics of included studies

A total of 32 studies, between 1981 and 2022, were included in the analysis. Many of the studies were conducted in the United States and Europe, with only a small number conducted in Japan and China. The diagnostic criteria used for ALS diagnosis in most studies were either the El Escorial Criteria or the revised El Escorial Criteria. All case-control studies were age and sex matched. In cohort studies, ALS confirmation was done through national registries, and the follow-up period ranged from 7 to 28 years. The characteristics of the included studies are summarized in Table 1 (the corresponding full references of the articles are given in Supplementary file Table 4).

### The pooled odds ratio of smoking and ALS

**Figure 2:**
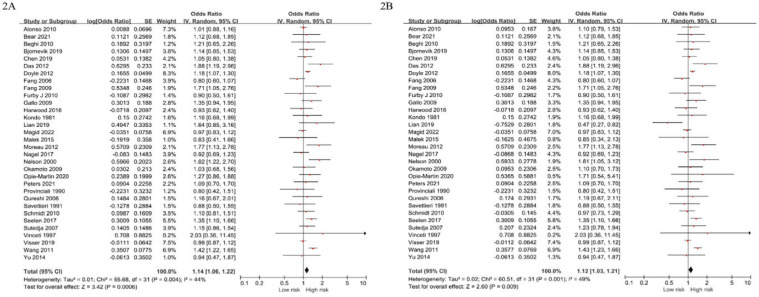
Forest plot of pooled odds ratios for the risk of amyotrophic lateral sclerosis in smoking group compared to the control group (2A: unadjusted, 2B: adjusted)

The present meta-analysis included 32 studies comprising 28 case-control studies and 4 cohort studies, from which the pooled OR of smoking and ALS was derived. The unadjusted OR was 1.14 (95% CI: 1.06–1.22, I^2^=44%, p<0.001), and the adjusted OR (AOR) was 1.12 (95% CI: 1.03–1.21, I^2^=49%, p=0.009) (Figure 2).

Additionally, the pooled OR between current smoking and ALS was derived from 22 studies, with an unadjusted OR of 1.28 (95% CI: 1.10–1.49, I^2^=66%, p<0.001) and an adjusted OR of 1.28 (95% CI: 1.10–1.48, I^2^=58%, p=0.001). Subgroup analysis by study design showed that the pooled OR of cohort studies was unadjusted 1.18 (95% CI: 0.96–1.44, I^2^=77%, p=0.11) and adjusted 1.18 (0.96-1.44, I^2^=77%, p=0.11), while that of case-control studies was unadjusted 1.12 (95% CI: 1.03–1.21, I^2^=28%, p=0.005) and adjusted 1.10 (95% CI: 1.00–1.20, I^2^=34%, p=0.05). Furthermore, subgroup analysis by sex revealed an unadjusted OR of 1.02 (95% CI: 0.85–1.22, I^2^=49%, p=0.84) and AOR of 1.01 (95% CI: 0.80–1.28, I^2^=58%, p=0.93) for men, whereas an unadjusted OR of 1.20 (95% CI: 1.10–1.30, I^2^=0%, p<0.001) and AOR of 1.25 (95% CI: 1.11–1.42, I^2^=11%, p<0.001) for women. The main and subgroup analysis results are presented in Table 2.

**Table 2:** Association between smoking and amyotrophic lateral sclerosis through main and subgroup analyses of included studies

*Outcome*	*Number of studies n*	*Heterogeneity %*	*OR (95% CI)*	*p*
**Smoking status**				
**Current and past smoking**				
Unadjusted	32	44	1.14 (1.06–1.22)	<0.001
Adjusted	32	49	1.12 (1.03–1.21)	0.009
**Current smoking**				
Unadjusted	22	66	1.28 (1.10–1.49)	<0.001
Adjusted	22	58	1.28 (1.10–1.48)	0.001
**Study design**				
**Case-control**				
Unadjusted	28	28	1.12 (1.03–1.21)	0.005
Adjusted	28	34	1.10 (1.00–1.20)	0.05
**Cohort**				
Unadjusted	4	77	1.18 (0.96–1.44)	0.11
Adjusted	4	77	1.18 (0.96–1.44)	0.11
**Gender**				
**Men**				
Unadjusted	7	49	1.02 (0.85–1.22)	0.84
Adjusted	7	58	1.01 (0.80–1.28)	0.93
**Women**				
Unadjusted	8	0	1.20 (1.10–1.30)	<0.001
Adjusted	8	11	1.25 (1.11–1.42)	<0.001

The subgroup difference In AOR based on sex (men, women) gave p=0.11, indicating no significant difference between these groups. In contrast, the subgroup difference in AOR based on study design (cohort, case-control) gave p=0.01, signifying a significant difference between these groups.

### Dose-response analysis between smoking (pack-years) and ALS

**Figure 3:**
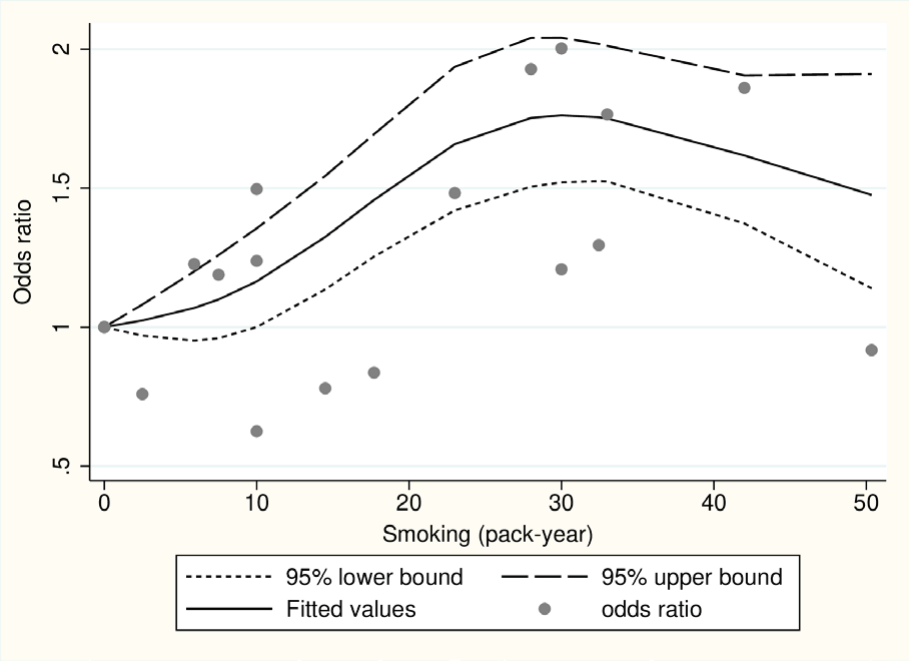
Dose-response graph between smoking and amyotrophic lateral sclerosis for five studies (two cohort and three case-control) using restricted cubic-spline analysis [x-axis: smoking (pack-years), y-axis: odds ratio of amyotrophic lateral sclerosis]

We performed a dose-response analysis on five studies (two cohort and three case-control) that provided pack-years information of smoking. We used the Wald test to assess linearity, which yielded a significant p=0.005. As a result, the assumption of linearity was rejected, suggesting that a non-linear model may be more suitable for predicting dose-response relationships. Figure 3 illustrates the dose-response graphs for non-linear models, indicating an inverted U-shaped curve.

### Risk of bias within studies

Among 28 case-control studies, 8 were rated as ‘good’ and 20 as ‘poor’. Of the 4 cohort studies, 2 were rated as ‘good’ and 2 as ‘fair’. The prevalent rating of ‘poor’ among the case-control studies is attributed to the fact that the assessment of exposure was not based on medical records or blinded interviews, and the response rate was either not mentioned or inconsistent between case and control group. A detailed evaluation of risk of bias is shown in Supplementary file Tables 2 and 3.

**Table 3:** GRADE approach for certainty assessment of overall analysis between smoking and amyotrophic lateral sclerosis

*Outcomes*	*Certainty assessment*							*Effect*	*Certainty*
	*Number of studies*	*Study design*	*Inconsistency*	*Indirectness*	*Imprecision*	*Publication bias*	*Other considerations*	*OR (95% CI)*	
Smoking – ALS	32	Serious*	Not serious**	Not serious	Not serious***	Not serious****	Dose-response gradient, residual confounding, or biases	1.14 (1.06–1.22)	Low

ALS: amyotrophic lateral sclerosis.

* All included studies are of observational design.

** Heterogeneity was 44%.

*** Very large samples size (over 4000) and p<0.05.

**** According to Egger’s regression test (p=0.504).

### Publication bias

**Figure 4:**
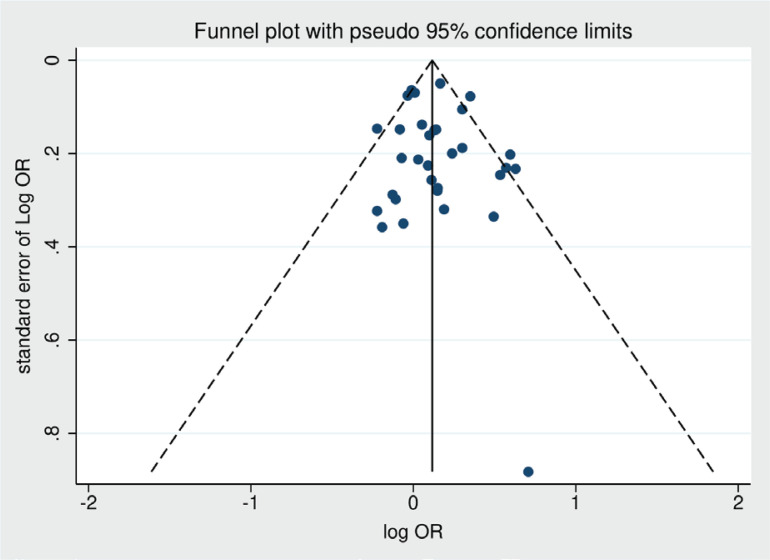
Funnel plot for evaluating the publishing bias of unadjusted pooled odds ratio derived from 32 studies (x-axis: log odds ratio, y-axis: standard error of log odds ratio)

Funnel plot was drawn to evaluate potential publication bias in the unadjusted OR results for smoking and ALS (Figure 4). The funnel plot did not reveal any significant publication bias. Additionally, Egge’s regression test was conducted and demonstrated no significant publication bias (p=0.504).

### Certainty assessment

A comprehensive evaluation of eight domains was conducted to assess the strength. To determine the strength of the primary outcome, a thorough assessment of eight domains was performed. The GRADE approach was utilized to evaluate the quality of evidence for the primary outcome, which was rated as low. A detailed evaluation of each domain is shown in Table 3.

## DISCUSSION

The primary finding of this study indicates a significant association between smoking and an increased risk of ALS, particularly in a dose-dependent manner. Previous studies have yielded inconsistent results regarding the relationship between smoking and ALS risk^19-22^. For instance, the Swedish Construction Workers Cohort, which involved 280558 male construction workers, did not find any evidence supporting an elevated risk of ALS associated with smoking^19^. A prior case-control study conducted in New England reported a weak association between smoking and ALS risk, but did not establish a dose-response relationship. Conversely, the European Prospective Investigation into Cancer and Nutrition (EPIC) cohort study, which examined mortality rates from ALS across different age groups, revealed that individuals who smoked for more than 33 years had a more than two-fold increased risk of developing ALS compared to those who never smoked^8^. The disparate findings among previous studies may be attributed to variations in study design, sample size, population characteristics, and methods employed to assess smoking exposure and ALS risk. The meta-analysis conducted by Alonso et al.^12^ does not offer strong support for a significant relationship between smoking and ALS risk, but rather hints at a possible association between smoking and an elevated risk of ALS in women. The inclusion of multiple studies and the utilization of a meta-analysis approach in our study address some of these limitations, resulting in a more comprehensive evaluation of the association. Our meta-analysis confirms the association between smoking and ALS risk. Furthermore, the dose-response curve exhibits an inverted U-shape. There is a limited number of studies conducted in the higher pack-years ranges of smoking, and this is believed to be influenced significantly by the single study results from Schmidt^8^, which show a low OR on the dose-response curve. It is anticipated that the statistical explanatory power of the dose-response relationship may be further strengthened as more studies accumulate in the future.

In our current analysis, we have identified a significant association between smoking and ALS in females, while no significant association was observed in males. Notably, a substantial proportion of ALS cases with bulbar onset were found in females, indicating that smoking may potentially act as a risk factor for bulbar ALS or contribute to an earlier onset of the disease in females^22^. A comprehensive analysis pooling data from cohort studies has indicated that smoking is a causal risk factor for ALS in females, and individuals with a history of smoking have a higher risk of developing ALS^23^. The absence of significant associations between smoking and ALS in males may be influenced by the fact that they are often more exposed to other potential risk factors for ALS, such as pesticides or organic solvents, during their occupational activities. This occupational confounding could have impacted the results in males. Therefore, further population-based studies specifically designed to investigate the causes of ALS are warranted. It is worth noting that many of the studies cited in this project were not originally designed with the specific aim of studying ALS etiology^8,23^.

While our study did not specifically investigate the mechanisms, we can propose hypotheses regarding the association between smoking and ALS. The association between smoking and ALS risk may be attributed to the potential impact of oxidization products from smoking on the impairment of mitochondria and endoplasmic reticulum^12,24^. Smoking introduces various oxidizing agents and toxic substances into the body, which can have detrimental effects on cellular components. Specifically, the oxidization products derived from smoking have been implicated in the impairment of mitochondria and endoplasmic reticulum^12,25^. Mitochondria are responsible for cellular energy production through oxidative phosphorylation. They play a crucial role in maintaining cellular homeostasis, including calcium regulation and reactive oxygen species (ROS) management. Oxidative stress induced by smoking can disrupt the normal functioning of mitochondria, leading to mitochondrial dysfunction. This dysfunction can result in increased ROS production, impaired energy production, and compromised cellular processes. The endoplasmic reticulum is a vital organelle involved in protein synthesis, folding, and calcium storage. Disruption of endoplasmic reticulum function can lead to the accumulation of misfolded proteins, endoplasmic reticulum stress, and activation of the unfolded protein response (UPR). The oxidizing agents present in cigarette smoke can induce endoplasmic reticulum stress, triggering the UPR and impairing the endoplasmic reticulum’s ability to properly fold and process proteins. These cellular dysfunctions, including mitochondrial dysfunction and endoplasmic reticulum stress, can initiate a cascade of events, including oxidative damage, inflammation, and neuronal death, which are hallmark features of ALS pathology^24^. While these proposed mechanisms provide a plausible explanation for the association between smoking and ALS, further research is necessary to fully elucidate the underlying molecular pathways and confirm these hypotheses.

### Limitations

Our study has several limitations that should be considered. Although we successfully established a dose-response relationship between smoking and the risk of ALS, it is important to note that our findings do not provide definitive evidence of a causal relationship. It is important to note that the majority of studies included in our analysis are case-control studies, which are susceptible to biases such as selection bias and confounding bias^28^. These biases have the potential to distort the results. Furthermore, it is difficult to differentiate between specific subtypes of ALS, such as sporadic ALS and familial ALS. The limited availability of individual-level data in the included studies prevented us from conducting subgroup analyses based on ALS subtypes. Furthermore, while we were able to estimate dose-response curves to a certain extent through restricted cubic-spline analysis, having individual-level data would allow for the generation of more precise dose-response curves. To address these limitations and provide more robust evidence, future studies should consider prospective designs, incorporate detailed information on potential confounders, and explore specific subtypes of ALS. Such efforts will contribute to a more comprehensive understanding of the association between smoking and ALS risk.

## CONCLUSIONS

Our study showed that there is a positive relationship between smoking and the risk of ALS. Furthermore, it revealed a significant association between smoking and ALS risk, particularly in women. To reduce the risk of developing ALS, it may be necessary to discontinue smoking, which is a modifiable risk factor.

## Data Availability

Data sharing is not applicable to this article as no new data were created.
