# Supplementary material for: Association of smoking with amyotrophic lateral sclerosis: A systematic review, meta-analysis, and dose-response analysis
Source: Tob Induc Dis. 2024 Jan 18;22:10.18332/tid/175731. doi: 10.18332/tid/175731 (PMC10795623; doi:10.18332/tid/175731)
Supplement: Supplementary file 1 [file TID-22-13-s1.pdf]

Supplementary Table 1. Search strategy for record extraction

Database	Search strategy
MEDLINE-Embase*	smok* OR tobacco OR nicoti* OR ciga* OR pipe<br><br>AND<br><br>amyotrophic lateral sclerosis OR als OR Gehrig* disease OR Lou-Gehrig* disease OR motor neuron disease OR Guam form OR Guam disease<br><br>AND<br><br>case control OR case base OR case referent OR case comparison OR cohort OR incidence
Web of Science	TI=(smok*) OR AB=(smok*) OR TI=(tobacco) OR AB=(tobacco) OR TI=(nicoti*) OR AB=(nicoti*) OR TI=(ciga*) OR AB=(ciga*) OR TI=(pipe) OR AB=(pipe)<br><br>AND<br><br>TI=(amyotrophic lateral sclerosis) OR AB=(amyotrophic lateral sclerosis) OR TI=(als) OR AB=(als) OR TI=(Gehrig* disease) OR AB=(Gehrig* disease) OR TI=(Lou-Gehrig* disease) OR AB=(Lou-Gehrig* disease) OR TI=(motor neuron disease) OR AB=(motor neuron disease) OR TI=(Guam form) OR AB=(Guam form) OR TI=(Guam disease) OR AB=(Guam disease)<br><br>AND<br><br>TI=(case control) OR AB=(case control) OR TI=(case base) OR AB=(case base) OR TI=(case referent) OR AB=(case referent) OR TI=(case comparison) OR AB=(case comparison) OR TI=(cohort) OR AB=(cohort) OR TI=(incidence) OR AB=(incidence)
Scopus	TITLE-ABS-KEY(smoke) OR TITLE-ABS-KEY(smoking) OR TITLE-ABS-KEY(smoker) OR TITLE-ABS-KEY(smokers) OR TITLE-ABS-KEY(tobacco) OR TITLE-ABS-KEY(nicotine) OR TITLE-ABS-KEY(cigarette) OR TITLE-ABS-KEY(pipe)<br><br>AND<br><br>TITLE-ABS-KEY(amyotrophic lateral sclerosis) OR TITLE-ABS-KEY(als) OR TITLE-ABS-KEY(Gehrig disease) OR TITLE-ABS-KEY (Gehrig's disease) OR TITLE-ABS-KEY(Lou-Gehrig disease) OR TITLE-ABS-KEY(Lou-Gehrig's disease) OR TITLE-ABS-KEY(motor neuron disease) OR TITLE-ABS-KEY(Guam form) OR TITLE-ABS-KEY(Guam disease)<br><br>AND<br><br>TITLE-ABS-KEY(case control) OR TITLE-ABS-KEY(case base) OR TITLE-ABS-KEY(case referent) OR TITLE-ABS-KEY(case comparison) OR TITLE-ABS-KEY(cohort) OR TITLE-ABS-KEY(incidence)

ScienceDirect	smoking OR smoker OR smokers<br><br>AND<br><br>amyotrophic lateral sclerosis OR ALS motor neuron disease<br><br>AND<br><br>case control OR cohort

* The Embase database was utilized for the search as it encompasses the search conducted in MEDLINE.

Supplementary Table 2. Risk of bias assessment for case-control studies – Newcastle-Ottawa Scale

Source	Selection				Comparability	Exposure			Assessment*
	**Is the case definition adequate?**	**Representativeness of cases**	**Selection of Controls**	**Definition of Controls**	**Comparability based on design and analysis**	**Assessment of exposure**	**Same method of ascertainment for cases and controls**	**Non-response rate**	
Kondo, 1981[1]		1			2		1		Poor
Provinciali, 1990[2]	1				2	1	1		Poor
Savettieri, 1991[3]	1	1	1	1	2		1		Poor
Vinceti, 1997[4]	1	1	1		2		1		Poor
Nelson, 2000[5]	1	1	1		2		1		Poor
Qureshi, 2006[6]	1	1	1	1	2		1		Poor
Sutedja, 2007[7]	1	1			2		1		Poor
Fang, 2009[8]	1	1	1	1	2		1		Poor
Okamoto, 2009[9]	1	1	1		2		1		Poor
Alonso, 2010[10]		1	1	1	2	1	1		Good
Beghi, 2010[11]	1	1	1		2		1		Poor
Furby J, 2010[12]	1	1		1	2	1	1		Good
Schmidt, 2010[13]	1	1	1	1	2	1	1		Good
Das, 2012[14]	1	1		1	2		1		Poor
Moreau, 2012[15]	1	1	1		2	1	1		Good
Yu, 2014[16]	1	1	1	1	2		1		Poor
Malek, 2015[17]	1	1			2		1		Poor
Harwood, 2016[18]	1	1		1	2	1	1		Good
Nagel, 2017[19]	1	1	1		2	1	1		Good
Seelen, 2017[20]	1	1	1	1	2		1		Poor
Bjornevik, 2019[21]	1	1	1		2	1	1		Good
Chen, 2019[22]		1	1	1	2		1		Poor
Lian, 2019[23]	1	1	1		2		1		Poor
Visser, 2019[24]	1	1	1		2		1		Poor

Opie-Martin, 2020[25]	1	1	1		2		1		Poor
Bear, 2021[26]		1	1	1	2		1		Poor
Peters, 2021[27]		1	1		2		1		Poor
Magid, 2022[28]		1	1	1	2	1	1		Good

* Good quality: 3 or 4 points in selection domain AND 1 or 2 points in compatibility domain AND 2 or 3 points in outcome/exposure domain, Fair quality: 2 points in selection domain AND 1 or 2 points in comparability domain AND 2 or 3 points in outcome/exposure domain, Poor quality: 0 or 1 point in selection domain OR 0 points in comparability domain OR 0 or 1 point in outcome/exposure domain

Supplementary Table 3. Risk of bias assessment for cohort studies – Newcastle–Ottawa Scale

Source	Selection				Comparability	Outcome			Assessment*
	Representativeness of the sample	Selection of the non-interventio n cohort	Ascertainment of exposure	Demonstration that outcome of interest was not present at start of study	Comparability based on design and analysis	Assessment of outcome	Was follow up long enough for outcomes to occur	Adequacy of follow-up of cohorts	
Fang, 2006[29]	1	1	1	1	1	1	1	1	Good
Gallo, 2009[30]	1	1			2	1	1	1	Fair
Wang, 2011[31]	1	1			2	1	1		Fair
Doyle, 2012[32]	1	1	1		1	1	1		Good

* Good quality: 3 or 4 points in selection domain AND 1 or 2 points in compatibility domain AND 2 or 3 points in outcome/exposure domain, Fair quality: 2 points in selection domain AND 1 or 2 points in comparability domain AND 2 or 3 points in outcome/exposure domain, Poor quality: 0 or 1 point in selection domain OR 0 points in comparability domain OR 0 or 1 point in outcome/exposure domain

Supplementary Table 4. Characteristics of included studies

	**Case-control studies**										
**No.**	**First Author Year**	**Location**	**Participants**	**Period of recruitment**	**Controls**	**Case ascertainment**	**DC**	**Smoking status**	**Cases Mean age (SD)**	**Controls Mean age (SD)**	**Matching**
1	Kondo 1981	Japan	158/158	1973	Community/ hospital	Neurology clinic	NS	Yes/No	NS	NS	Age, sex, residence
2	Provinciali 1990	Ancona Italy	77/80	1979–1987	Other neurological diseases	Neurology clinic	NS	10–30 cigarettes/day	59 (8)	57 (9)	Age, sex, regional origin, life-style, cultural background
3	Savettieri 1991	Palermo Italy	46/92	NS	Friends/neighbors	Neurology clinic	NS	Yes / No	NS	NS	Age, sex, residence, socioeconomic status
4	Vinceti 1997	Reggio Emilia Italy	16/39	NS	Community	ALS clinic	EEC	Yes/No	65.9 (14.0)	64.4 (12.9)	Age and sex
5	Nelson 2000	Washington State USA	161/321	1990–1994	Community	Multiple sources	NS	Never/ever/ former/ current	61.4 (1.0)	61.7 (0.7)	Age and sex
6	Qureshi 2006	Boston USA	95/106	1998–2002	Friends/relatives	ALS clinic	EEC	Yes/No	54.4 (13.1)	52.5 (14.9)	Age and sex
7	Sutedja 2007	Utrecht Netherlands	364/392	2001–2005	Friends	ALS clinic	EEC	Never/former/ current/	60.2 (11.7)	60.0 (10.9)	Age and sex

8	Fang 2009	New England USA	109/253	1993–1996	Community	Neurology clinic	EEC	0/1–10/ 11–30/31+ (pack-years)	NS	NS	Age, sex, residence
9	Okamot 2009	Tokai Japan	153/306	2000–2005	Community	Neurology clinic	EEC	Non-smoker/ current	63.7 (9.2)	63.4 (10.6)	Age, sex
10	Alonso 2010	UK	1143/11371	1990–2008	GPRD database	GPRD database	NA	Never/former/ current/ non-heavy/ heavy	67.4 (12.5)	67.1 (12.5)	Age, sex, practice, year of enrolment
11	Beghi 2010	EURALS Consortium (Italy, UK, Ireland)	61/112	NS	Community	ALS registries	EEC	Yes/No	63.7 (NS)	62.3 (NS)	Age and sex
12	Furby 2010	Brittany France	108/112	2006–2008	Hospital (orthopedic service for minor trauma)	Neurology clinic	EEC	Non-smoker/ former/ current/ pack-years	68 (18.0)	65 (18.0)	Age and sex
13	Schmidt 2010	USA	241/597	2003–2007	US army veterans	US army veterans ALS registry	EEC	Never/former/ current	62.4 (10.3)	61.7 (10.6)	Age, sex, use of veteran affairs health care
14	Das 2012	India	110/240	2008–2011	Community	Neurology clinic	rEEC	Non-present/ present	NS	NS	Age and sex
15	Moreau 2012	Nord Pas de Calais County France	102/408	2003–2009	Community	ALS clinic	EEC	Never/former/ current	NS	NS	Age and sex
16	Yu 2014	Michigan USA	66/66	NS	Community	ALS clinic	rEEC	Never/former/ current	NS	NS	Age and sex

17	Malek 2015	Pittsburgh and Philadelphia USA	66/66	2008–2010	Outpatient hospital controls	ALS clinic	EEC	Never/ever	57.1 (13.2)	56.4 (13.5)	Age, sex, race
18	Harwood 2016	Northern England UK	175/317	2009–2013	Community	Hospital / community	rEEC	Non-smoker/ ex-smoker/ current	64 (NS)	65 (NS)	Age and sex
19	Nagel 2017	South-West Germany	289/506	2010–2014	Community	ALS registry Swabia	rEEC	Never/ever	65.7 (10.5)	66.3 (9.8)	Age and sex
20	Seelen 2017	Netherlands	917/2662	2006–2013	Community	Multiple sources	rEEC	Non-current/ current	63.5	63.5	Age and sex
21	Bjornevik 2019	USA	275/549	1976–2012	Cohort	5 Cohort	rEEC	Never/past/ current	64.6 (7.2)	64.6 (7.2)	Age, sex, cohort, fasting status, time of blood draw
22	Chen 2019	New Zealand	321/605	2013–2016	Community	ALS registry and hospital discharge records	NS	Never/ ex-smoker/ current	NS	NS	Age and sex
23	Lian 2019	China	123/239	2013–2016	Community	Hospital	EEC	Never/former/ current	53.2 (9.6)	53.0 (11.1)	Age and sex
24	Visser 2019	Euro-MOTOR consortium (Netherlands, Ireland, Italy)	1577/2922	2011–2014	Community	Multiple sources	rEEC	Never/former/ current	NS	NS	Age, sex, residence
25	Opie-Martin	UK	202/200	2008–2013	Community	MNDA	EEC	Never/former/	63.1	64.5	Age and sex

	2020					Epidemiology study		current	(10.53)	(10.52)	
26	Bear<br>2021	USA	127/127	2018–2020	Community	National ALS Registry	NS	Never/ever/<br>current	NS	NS	Age, sex, residence
27	Peters<br>2021	Europe	107/319	1993–1999	Cohort	EPIC cohort	NS	Never/former/<br>current	60.5<br>(NS)	60.4<br>(NS)	Age, sex, study center
28	Magid<br>2022	USA	3714/18570	2006–2013	Cohort	Centers for Medicare and Medicaid Services (CMS)	EEC	Yes/No	75.7<br>(5.7)	75.7<br>(5.8)	Age, sex, enrollment length, residence
	**Cohort studies**										
	**First Author Year**	**Location**	**Participants**	**Period of recruitment**	**Average follow-up**	**Case ascertainment**	**DC**	**Smoking status**		**Age (range)**	**Adjustment**
29	Fang<br>2006	Sweden	160/280558	1978–1983	19.6 years	Inpatient register	NS	Non-tobacco use/former/<br>current		41<br>(NS)	Age, residence
30	Gallo<br>2009	Europe	116/505355	1991–2001	8.9 years	Death certificates	NA	Never/former/<br>current		51<br>(NS)	Age, sex, education level, study center
31	Wang<br>2011	USA	816/1119080	1986–2005	7–28 years	US NDI/self-report	NS	Never/ever/<br>former/<br>current		NS	Age, sex, body mass index, physical activity, education level
32	Doyle<br>2012	UK	752/1319360	1981–2008	9.2 years	ICD-10	NS	Never/past/<br>current		56	Region, socioeconomic status, year of birth, body mass index, use

											of hormone replacement therapy, smoking, alcohol use, as appropriate

DC: Diagnostic criteria. ALS: amyotrophic lateral sclerosis. EEC: El Escorial Criteria. NA: not applicable. NS: not specified. NDI: National Death Index. rEEC: revised El Escorial Criteria (Airlie House Criteria). SES: socioeconomic status.

References

1. Kondo K, Tsubaki T. Case-control studies of motor neuron disease: association with mechanical injuries. *Arch Neurol* 1981;38(4):220-226. doi:10.1001/archneur.1981.00510040046007
2. Provinciali L, Giovagnoli AR. Antecedent events in amyotrophic lateral sclerosis: do they influence clinical onset and progression? *Neuroepidemiology* 1990;9(5):255-262. doi:10.1159/000110782
3. Savettieri G, Salemi G, Arcara A, et al. A case-control study of amyotrophic lateral sclerosis. *Neuroepidemiology* 1991;10(5-6):242-245. doi:10.1159/000110279
4. Vinceti M, Guidetti D, Bergomi M, et al. Lead, cadmium, and selenium in the blood of patients with sporadic amyotrophic lateral sclerosis. *Ital J Neurol Sci* 1997;18:87-92. doi:10.1007/BF01999568
5. Nelson LM, McGuire V, Longstreth Jr W, et al. Population-based case-control study of amyotrophic lateral sclerosis in western Washington State. I. Cigarette smoking and alcohol consumption. *Am J Epidemiol* 2000;151(2):156-163. doi:10.1093/oxfordjournals.aje.a010184
6. Muddasir Qureshi M, Hayden D, Urbinelli L, et al. Analysis of factors that modify susceptibility and rate of progression in amyotrophic lateral sclerosis (ALS). *Amyotroph Lateral Sc* 2006;7(3):173-182. doi:10.1080/14660820600640596
7. Sutedja N, Veldink J, Fischer K, et al. Lifetime occupation, education, smoking, and risk of ALS. *Neurology* 2007;69(15):1508-1514. doi:10.1212/01.wnl.0000277463.87361.8c
8. Fang F, Quinlan P, Ye W, et al. Workplace exposures and the risk of amyotrophic lateral sclerosis. *Environ Health Perspect* 2009;117(9):1387-1392. doi:10.1289/ehp.0900580
9. Okamoto K, Kihira T, Kondo T, et al. Lifestyle factors and risk of amyotrophic lateral sclerosis: a case-control study in Japan. *Ann Epidemiol* 2009;19(6):359-364. doi:10.1016/j.annepidem.2009.01.015
10. Alonso A, Logroscino G, Jick SS, et al. Association of smoking with amyotrophic lateral sclerosis risk and survival in men and women: a prospective study. *BMC Neurol* 2010;10:1-6. doi:10.1186/1471-2377-10-6
11. Beghi E, Logroscino G, Chiò A, et al. Amyotrophic lateral sclerosis, physical exercise, trauma and sports: results of a population-based pilot case-control study. *Amyotroph Lateral Sc* 2010;11(3):289-292. doi:10.3109/17482960903384283
12. Furby A, Beauvais K, Kolev I, et al. Rural environment and risk factors of amyotrophic lateral sclerosis: a case–control study. *J Neurol* 2010;257(5):792-798. doi:10.1007/s00415-009-5419-5
13. Schmidt S, Kwee LC, Allen KD, et al. Association of ALS with head injury, cigarette smoking and APOE genotypes. *J Neurol Sci* 2010;291(1-2):22-29.

doi:10.1016/j.jns.2010.01.011
14. Das K, Nag C, Ghosh M. Familial, environmental, and occupational risk factors in development of amyotrophic lateral sclerosis. *N Am J Med Sci* 2012;4(8):350. doi:10.4103/1947-2714.99517
15. Moreau C, Brunaud-Danel V, Dallongeville J, et al. Modifying effect of arterial hypertension on amyotrophic lateral sclerosis. *Amyotroph Lateral Sc* 2012;13(2):194-201. doi:10.3109/17482968.2011.610110
16. Yu Y, Su F-C, Callaghan BC, et al. Environmental risk factors and amyotrophic lateral sclerosis (ALS): a case-control study of ALS in Michigan. *PLoS One* 2014;9(6):e101186. doi:10.1371/journal.pone.0101186
17. Malek AM, Barchowsky A, Bowser R, et al. Exposure to hazardous air pollutants and the risk of amyotrophic lateral sclerosis. *Environ Pollut* 2015;197:181-186. doi:10.1016/j.envpol.2014
18. Harwood CA, Westgate K, Gunstone S, et al. Long-term physical activity: an exogenous risk factor for sporadic amyotrophic lateral sclerosis? *Amyotroph Lateral Scler Frontotemporal Degener* 2016;17(5-6):377-384. doi:10.3109/21678421.2016.1154575
19. Nagel G, Peter RS, Rosenbohm A, et al. Adipokines, C-reactive protein and Amyotrophic Lateral Sclerosis–results from a population-based ALS registry in Germany. *Sci Rep* 2017;7(1):1-9. doi:10.1038/s41598-017-04706-5
20. Seelen M, Toro Campos RA, Veldink JH, et al. Long-term air pollution exposure and amyotrophic lateral sclerosis in Netherlands: a population-based case–control study. *Environ Health Perspect* 2017;125(9):097023. doi:10.1289/EHP1115
21. Bjornevik K, O'Reilly ÉJ, Berry JD, et al. Prediagnostic plasma branched-chain amino acids and the risk of amyotrophic lateral sclerosis. *Neurology* 2019;92(18):e2081-e88. doi:10.1212/WNL.0000000000006669
22. Chen GX, Martine't Mannetje A, Douwes J, et al. Occupation and motor neuron disease: a New Zealand case–control study. *Occup Environ Med* 2019;76(5):309-316. doi:10.1136/oemed-2018-105605
23. Lian L, Liu M, Cui L, et al. Environmental risk factors and amyotrophic lateral sclerosis (ALS): a case-control study of ALS in China. *J Clin Neurosci* 2019;66:12-18. doi:10.1016/j.jocn.2019.05.036
24. Visser AE, D'Ovidio F, Peters S, et al. Multicentre, population-based, case–control study of particulates, combustion products and amyotrophic lateral sclerosis risk. *J Neurol Neurosurg Psychiatry* 2019;90(8):854-860. doi:10.1136/jnnp-2018-319779
25. Opie-Martin S, Jones A, Iacoangeli A, et al. UK case control study of smoking and risk of amyotrophic lateral sclerosis. *Amyotroph Lateral Scler Frontotemporal Degener* 2020;21(3-4):222-227. doi:10.1080/21678421.2019.1706580
26. Bear TM, Malek AM, Foulds A, et al. Recruitment of population-based controls for ALS cases from the National ALS Registry. *Amyotroph Lateral Scler Frontotemporal Degener* 2021;22(5-6):395-400. doi:10.1080/21678421.2021.1887262
27. Peters S, Broberg K, Gallo V, et al. Blood metal levels and amyotrophic lateral sclerosis risk: a prospective cohort. *Ann Neurol* 2021;89(1):125-133. doi:10.1002/ana.25932
28. Magid HSA, Topol B, McGuire V, et al. Cardiovascular Diseases, Medications, and ALS: A Population-Based Case-Control Study. *Neuroepidemiology* 2022:1-10. doi:10.1159/000526982
29. Fang F, Bellocco R, Hernán MA, et al. Smoking, snuff dipping and the risk of amyotrophic lateral sclerosis–a prospective cohort study. *Neuroepidemiology* 2006;27(4):217-221. doi:10.1159/000096956
30. Gallo V, Bueno-De-Mesquita HB, Vermeulen R, et al. Smoking and risk for amyotrophic lateral sclerosis: analysis of the EPIC cohort. *Ann Neurol* 2009;65(4):378-385. doi:10.1002/ana.21653
31. Wang H, O'Reilly ÉJ, Weisskopf MG, et al. Smoking and risk of amyotrophic lateral sclerosis: a pooled analysis of 5 prospective cohorts. *Arch Neurol* 2011;68(2):207-213. doi:10.1001/archneurol.2010.367
32. Doyle P, Brown A, Beral V, et al. Incidence of and risk factors for motor neurone disease in UK women: a prospective study. *BMC Neurol* 2012;12:1-7. doi:10.1186/1471-2377-12-25

Supplementary Table 5. Excluded studies and reasons for exclusion

Title	DOI	Reason
Longitudinal comparison of the self-entry Amyotrophic Lateral Sclerosis Functional Rating Scale-Revised (ALSFRS-RSE) and Rasch-Built Overall Amyotrophic Lateral Sclerosis Disability Scale (ROADS) as outcome measures in people with amyotrophic lateral sclerosis	10.1002/mus.27691	No control group
Association between DNA methylation variability and self-reported exposure to heavy metals	10.1038/s41598-022-13892-w	No matching
Occupational lead exposure and survival with amyotrophic lateral sclerosis	10.1080/21678421.2022.2059379	No control group
Association between vascular risk factors and cognitive impairment in amyotrophic lateral sclerosis: a case-control study	10.1080/21678421.2022.2108327	No control group
Low incidence of advanced neurological burden but high incidence of age-related conditions that are dementia risk factors in aging people living with HIV: a data-linkage 10-year follow-up study	10.1007/s13365-022-01104-0	Study that did not report the outcome of interest
An amyotrophic lateral sclerosis hot spot in the French Alps associated with genotoxic fungi	10.1016/j.jns.2021.117558	No available data
D-amino acid oxidase (DAO) rare genetic missense variant p.Pro103Leu and gastric cancer	10.3892/mco.2021.2220	Study that did not report the outcome of interest
Data mining analysis of demographic and clinical factors in turkish amyotrophic lateral sclerosis patients	10.4103/NSN.NSN_69_20	No matching
Occupation and amyotrophic lateral sclerosis risk: a case-control study in the isolated island population of Malta	10.1080/21678421.2021.1905847	No available data
Finding Waldo in Narcosis: Identifying Respiratory-Onset Motor Neuron Disease in a Chronic Smoker With Triple-Vessel Coronary Heart Disease	10.1016/j.amjmed.2020.05.041	Case report
Liver injury risk factors in amyotrophic lateral sclerosis patients treated with riluzole	10.1248/yakushi.20-00015	No control group
Is the incidence of motor neuron disease higher in French military personnel?	10.1080/21678421.2019.1675709	No available data
Influence of environment and lifestyle on incidence and progress of amyotrophic lateral sclerosis in A German ALS population	10.14336/AD.2018.0327	No matching
Relationship of statins and other cholesterol-lowering medications and risk of amyotrophic lateral sclerosis in the US elderly	10.1080/21678421.2018.1511731	No available data
Clinical and prognostic features of ALS/MND in different phenotypes-data from a hospital-based registry	10.1016/j.brainresbull.2018.09.005	No control group
Prediagnostic body size and risk of amyotrophic lateral sclerosis death in 10 studies	10.1080/21678421.2018.1452944	No available data
Association of serum retinol-binding protein 4 concentration with risk for and prognosis of amyotrophic lateral sclerosis	10.1001/jamaneurol.2017.5129	No available data
Phenotypic differences of amyotrophic lateral sclerosis (ALS) in China and Germany	10.1007/s00415-018-8735-9	No control group
Myasthenia gravis seronegative for acetylcholine receptor antibodies in South Korea: Autoantibody profiles and clinical features	10.1371/journal.pone.0193723	Study that did not report the outcome of interest
The incidence of depression among residents of assisted living: Prevalence and related risk factors	10.2147/CIA.S147436	Study that did not report the outcome of interest
Cardiometabolic health and risk of amyotrophic lateral sclerosis	10.1002/mus.25547	No control group
A case-control study of hormonal exposures as etiologic factors for ALS in women	10.1212/WNL.0000000000004390	No available data
Farming and incidence of motor neuron disease: French nationwide study	10.1111/ene.13353	Study that did not report the outcome of interest
Medical history of chemotherapy or immunosuppressive drug treatment and risk of amyotrophic lateral sclerosis (ALS)	10.1007/s00415-017-8564-2	No matching
Environmental and Occupational Exposures and Amyotrophic Lateral Sclerosis in New England	10.1159/000453359	No matching
Cardiovascular disease and diagnosis of amyotrophic lateral sclerosis: A population based study	10.1080/21678421.2016.1208247	No available data
Accrued somatic mutations (nucleic acid changes) trigger ALS: 2005-2015 update	10.1002/mus.25049	Review paper
Physical activity and risk of Amyotrophic Lateral Sclerosis in a prospective cohort study	10.1007/s10654-016-0119-9	No available data
Association of alcohol use disorders with amyotrophic lateral sclerosis: A Swedish national cohort study	10.1111/ene.12667	No available data
Is the risk of motor neuron disease increased or decreased after cancer? An Australian case-control study	10.1371/journal.pone.0103572	No available data
To what degree is the association between educational inequality and laryngeal cancer explained by smoking, alcohol consumption, and occupational exposure?	10.5271/sjweh.3403	Study that did not report the outcome of interest
Vitamin E serum levels and controlled supplementation and risk of amyotrophic lateral sclerosis	10.3109/21678421.2012.745570	No available data

Current pathways for epidemiological research in amyotrophic lateral sclerosis	10.3109/21678421.2013.778565	Review paper
Premorbid body mass index and risk of amyotrophic lateral sclerosis	10.3109/21678421.2012.735240	No available data
Replication of association of CHRNA4 rare variants with sporadic amyotrophic lateral sclerosis: The Italian multicentre study	10.3109/17482968.2012.704926	Study that did not report the outcome of interest
Smoking is not a risk factor for sporadic amyotrophic lateral sclerosis in an Australian population	10.1159/000336013	No matching
Cancer in patients with motor neuron disease, multiple sclerosis and Parkinson's disease: Record linkage studies	10.1136/jnnp.2009.175463	Study that did not report the outcome of interest
An exploratory case-control study on spinal and bulbar forms of amyotrophic lateral sclerosis in the province of Rome	10.3109/17482960802382313	No matching
Paraneoplastic encephalomyelitis associated with motor neuron disease causing respiratory failure in the setting of occult small cell lung carcinoma	10.1111/j.1743-7563.2008.00155.x	Study that did not report the outcome of interest
Increase in mortality for motor neuron disease in Italy, 1980-1999	NS	No available data
Acquired nucleic acid changes may trigger sporadic amyotrophic lateral sclerosis	10.1002/mus.20372	Review paper
Associations of insulin-like growth factors, insulin-like growth factor binding proteins and acid-labile subunit with coronary heart disease	10.1111/j.1365-2265.2004.02136.x	Study that did not report the outcome of interest
Genotoxic effects of occupational exposure to lead and cadmium	10.1016/s1383-5718(03)00167-0	Study that did not report the outcome of interest
Inter- and intraindividual variability of riluzole serum concentrations in patients with ALS	10.1016/s0022-510x(01)00613-x	No control group
Coughing and choking in motor neuron disease	10.1136/jnnp.68.5.601	No available data
Cholinergic receptors in cognitive disorders	10.1017/s0317167100037240	Study that did not report the outcome of interest
Neurocognitive health of older adults experiencing homelessness in Oakland, California	10.3389/fneur.2022.905779	Cross-sectional design
Study on the Mechanism of Astragalus Polysaccharide in Treating Pulmonary Fibrosis Based on “Drug-Target-Pathway” Network	10.3389/fphar.2022.865065	Review paper
Assessing lifestyle behaviours of people living with neurological conditions: A panoramic view of community dwelling australians from 2007–2018	10.3390/jpm11020144	Study that did not report the outcome of interest
Alcohol Drinking and Amyotrophic Lateral Sclerosis: An Instrumental Variable Causal Inference	10.1002/ana.25721	Mendelian randomization study
Epidemiology of amyotrophic lateral sclerosis	10.1016/B978-0-12-802973-2.00013-6	Review paper
Preliminary results of national Amyotrophic Lateral Sclerosis (ALS) registry risk factor survey data	10.1371/journal.pone.0153683	Cross-sectional design
Epidemiology of amyotrophic lateral sclerosis: A review of literature	10.1016/j.neurol.2015.11.002	Review paper
Motor neurone disease and military service: Evidence from the Scottish Veterans Health Study	10.1136/oemed-2015-103066	Study that did not report the outcome of interest
Is exposure to cyanobacteria an environmental risk factor for amyotrophic lateral sclerosis and other neurodegenerative diseases?	10.3109/21678421.2012.750364	Review paper
Earlier onset and shorter survival of amyotrophic lateral sclerosis in Jewish patients of North African origin. A clue to modifying genetic factors?	10.1016/j.jns.2007.02.021	No control group
Motor neurone disease in the Lothian Region of Scotland 1961-81	1961-81<br>10.1136/jech.40.4.344	No control group
Epidemiology of Major Neurodegenerative Diseases in Women: Contribution of the Nurses' Health Study	10.2105/AJPH.2016.303324	Review paper

*NS, not specified

© 2024 Kim K. et al.
